# Wetting ability of an acetone/based etch&rinse adhesive after NaOCl-treatment

**DOI:** 10.4317/medoral.17654

**Published:** 2012-02-09

**Authors:** Fátima S. Aguilera, Raquel Osorio, Estrella Osorio, Pedro Moura, Manuel Toledano

**Affiliations:** 1 Department of Dental Materials, School of Dentistry, Campus de Cartuja 18071, University of Granada, Spain; 2 Department of Restorative Dentistry, Instituto Superior de Ciências da Saúde-Sul Egas Moniz, Campus Universitario Quinta da Granja, Monte da Caparica 2829-511, Portugal

## Abstract

Objectives: to evaluate the effect of sodium hypochlorite (NaOCl) treatment on surface dentin roughness (Ra) and contact angle (CA) when using Prime&Bond NT adhesive (PB NT). Study Design: Extracted human third molars were sectioned to expose flat, superficial and deep dentin surfaces. CA and Ra were measured (1) before and (2) after 35% H3PO4 etching, and (3) H3PO4 etching + 5% NaOCl treated for 2 minutes before the application of PB NT. CA was measured by the Axisymmetric Drop Shape Analysis Technique using distilled and deionized water and PB NT. Roughness was evaluated with a profilometer, twelve radial measurements were performed in each treatment surface. Data were analyzed with two-way ANOVA and Newman-Keuls multiple comparison test procedures. Results: CA values decreased after acid etching and even more after NaOCl treatment on deep dentin when water was tested. With resin, there were not differences on CA results after H3PO4 neither after NaOCl treatment, in both dentin surfaces. Etching and NaOCl treatment resulted in surface roughness increase. Conclusions: In spite of the higher roughness after NaOCl treatment on superficial and deep dentin, the use of 5% NaOCl for 2 min after dentin demineralization when PB NT was employed did not improved the wettability of dentin, probably due to nanofiller content and/or hydrogen-bonding interactions with residues of the organic matrix on collagen-depleted dentin.

** Key words:**Sodium hypochlorite, contact angle, roughness, Prime&Bond NT, superficial dentin, deep dentin.

## Introduction

Acid etching of dentin is used as a surface preparation step to improve adhesion for a variety of procedures in restorative dentistry. After etching, hydroxyapatite is removed, the hydrated intertubular collagen network is exposed and hydrophilic adhesives are able to penetrate this space and form the so called hybrid layer (1). Several factors influence on biodegradation of the collagen matrix and/or hydrophilic resin components within the hybrid layer. Some of them are incomplete penetration/infiltration of resin into dentin substrate (2) and hydrolysis of unprotected collagen (3,4), resulting in continued degradation of resindentin interface (2). To avoid this biodegradation different strategies have been proposed, such as the use of metalloproteinases inhibitors (5) and demineralized collagen removal (6).

Sodium hypochlorite (NaOCl) is a nonspecific proteolytic agent that effectively removing organic components at room temperature (7). The literature shows indeed that NaOCl treatment of aciddemineralized dentin has been advocated as an intermediate conditioning step, capable of removing the exposed surface collagen and producing protein-depleted channels into intact subsurface dentin (8,9). Several researchers have studied the role of NaOCl in dentin adhesion (8,9). Thus, the contribution of collagen should be studied for different bonding systems and from different approaches based on technology.

The success of the bond between adhesive resin and dentin depends (1) on the penetration of the primer and the adhesive resin into the conditioned dentin surface (10) and (2) on the superficial adhesion by contact with the adhesive (11). As for these mechanisms, wettability of the surface is the first requirement; an effective characterization of the latter should be of great utility for the design of the adhesive systems (12). Wettability is strongly dependent on roughness, chemical composition, and hydration state and could be influenced by numerical tubule density. Contact angle measurements, being a popular technique since they provide information about wettability, are a noninvasive manner (12). Therefore, changes in the superficial and deep dentin structure resulting from etching and NaOCl treatment could influence wettability of adhesive system (13).

This in vitro study was conducted to determine the effect of 5% aqueous NaOCl solution on contact angle (CA) measurements and roughness (Ra) of a one-bottle dentin adhesive system containing acetone as solvent. The null hypothesis tested was that phosphoric acid and sodium hypochlorite pretreatments do not influence these surface properties of an acetone/based etch&rinse adhesive to superficial and deep dentin.

## Material and Methods

-Specimen preparation

Twenty caries-free extracted human third molars were stored in 0.5% chloramine T (Sigma–Aldrich, S.A., Madrid, Spain) at 4oC for up to 1 month were used, as ISO standard 11405 recommends (14). Human specimens were obtained with the informed consent of donors, under a protocol that was reviewed and approved by the Institutional Ethics Committee. The teeth were cleaned of debris and mounted in phenolic rings with cold-cured acrylic resin, leaving the occlusal two-thirds of the crown exposed. The specimens were sectioned below the dentinenamel junction ground flat and automatically polished up to 600-grit (Struers LaboPol-4, Struers, Copenhagen, Denmark) using silicon carbide papers under running water to provide flat dentin surfaces.

-Contact angle (CA) measurement

The specimens were randomly assigned to two equal groups (n=10). The ADSA-CD technique (Axisymmetric Drop Shape Analysis - Contact Diameter) (15) was employed for contact angle measurements. The groups and procedure were as exposed in Toledano et al (13), using water and Prime & Bond NT dental adhesive on superficial and deep etched or etched and NaOClaq solution (5%) treated dentin.

The hydration state of dentin was carefully controlled. Just before the contact angle measurements, excess water was removed from the surface, avoiding desiccating the dentin, leaving a moist and slightly glossy surface. However, no excess water remained on the dentin surface. All contact angle measurements were made into a thermostatic cell at 25º C and close to humidity saturation. Data were obtained in degrees.

-Microroughness measurements (Ra)

Surface roughness of each sample was measured by a conventional diamond stylus profilometer (Mitutoyo Surftest 201, Tokyo, Japan) after performing the water contact angle measurements in all groups. The radius of the tip was 5 µm, and the tip pressure of 4 mN. For each sample, twelve radial measurements of 2.25 mm (traversing length) were made, that included 1 mm for the preliminary run and 1.25 mm for the evaluation length. Vertical movement of the stylus was recorded as a varying output voltage that was digitized by a minicomputer. The Ra (arithmetic mean deviation of the roughness profile) was calculated in micrometres. The hydration state of dentin was controlled as explained above.

-Statistical analysis

Contact angle observed (º) and microroughness values (Ra –nm-) were analyzed by ANOVA (dentin depth and dentin treatment as independent variables) and Student Newman Keuls multiple comparisons tests. Statistical significance was set in advance at the 0.05 probability level.

## Results

 Table 1 display means and standard deviations for contact angle (º) obtained on superficial and on deep dentin. Two-way ANOVA indicated that surface dentin treatment (F=27.42; p<0.001) and dentin depth (F=5.71; p<0.01) had a statistically significant effect on the water observed contact angle. The observed contact angle when measured with water decreased on acid-etched dentin as compared to ground dentin (covered with smear layer), regardless of dentin depth. Mean water contact angle obtained on etched vs. etched and NaOCl treated dentin was statistically similar for superficial dentin, but different for deep dentin.

**Table 1:**
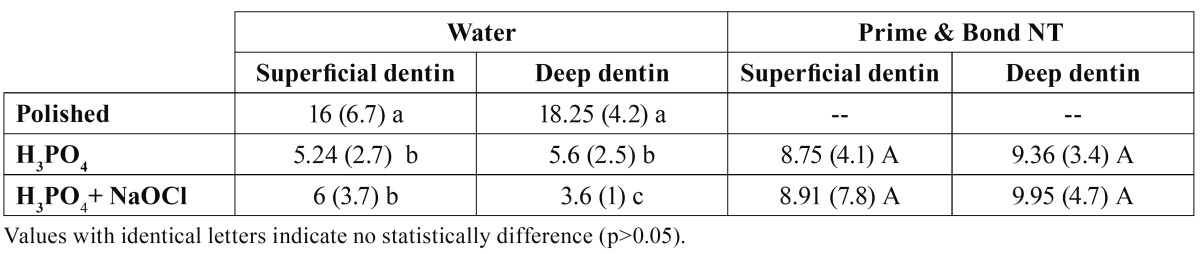
Mean (SD) of water and Prime&Bond NT observed contact angle values (degrees) on superficial and deep dentin after acid etching and acid/NaOCl application.

PB NT observed contact angles were not affected neither by dentin depth (P=0.97) nor NaOCLaq treatment surface (P=0.85).

 Table 2 shows means and standard deviations for average roughness, Ra (µm), as measured with the profilometer on superficial and deep dentin. ANOVA found that surface dentin treatment had a significant effect (F=7.95; p<0.001) on dentin roughness, but dentin depth did not result in significant differences (p=0.38). Dentin etching increased surface roughness on deep dentin, but not on superficial dentin. When superficial and deep dentin were etched and NaOClaq treated, dentin roughness increased significantly.

**Table 2:**
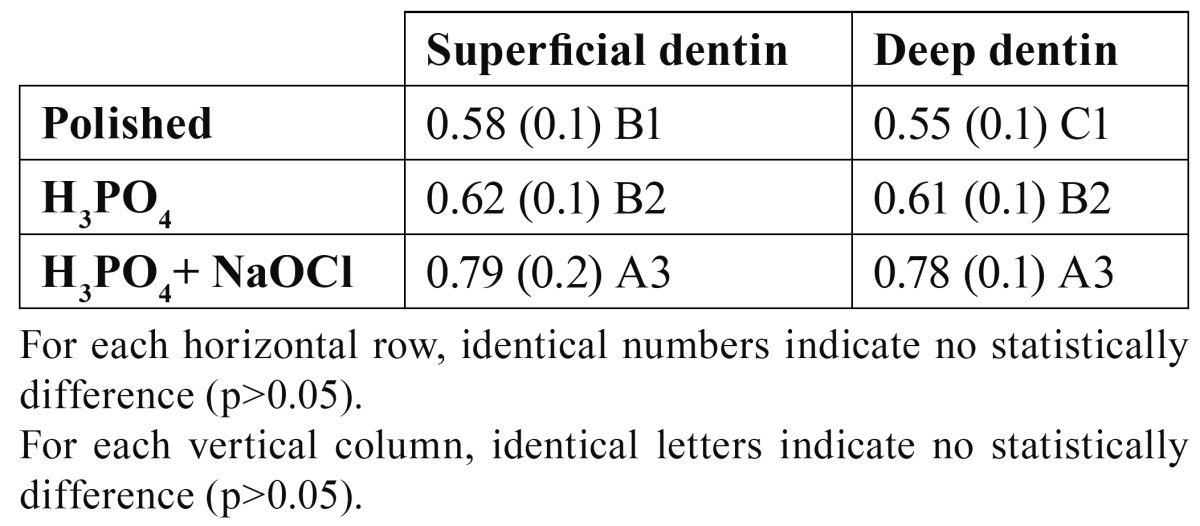
Mean (SD) surface roughness (µm) on superficial and deep dentin after acid etching and acid etching/NaOCl application.

## Discussion

Wettability is one of the most important physicochemical surface properties. Contact angle measurements determine the wettability of the substratum (16,17). Low contact angles are an indication of high surface free energy, whereas high contact angles indicate low surface free energy. The surface tension of the adhesive must be as low possible, while the substrate surface energy must be as high as possible (16).

Dentin is a dynamic substrate with a complex organic structure and biological activity that preclude the establishment of a reliable and durable bonding (18). Dentin is composed of two different substrates, collagen which has a low surface energy, and hydroxyapatite, wich has a high surface energy (8). Superficial and deep dentin have been studied separately to determine the influence of tubule numerical density on roughness and wettability. In the present study, a significant increase in wettability (decrease in mean water observed contact angle) was found on both superficial and deep dentin after acid etching, being in agreement with other authors (12). After acid etching, the surface energy is decreased by the exposures of collagen fibers and loss of mineral component (13). When both dentins were etched, smear layer disappeared, the morphological characteristics of dentin was shown (19) and roughness increased (13), but only significantly on deep dentin ( Table 2). Thus, intertubular dentin is eroded and contributes to increase the roughness (10). In addition, the tubules lost the peritubular dentin assuming funnel shape (13). This increases the chemical homogeneity of the surface and, above all, unblocks the tubules occluded by the smear layer, augmenting roughness and porosity (12). Following Wenzel equation, the irregularities of the solid surface influenced contact angle measurements and expressed these findings as follows (20): r = cos θ1/θ2 in which r gives the ratio of actual to apparent or projected area, and contact angles θ1 and θ2 refer to the roughened surface and true (smooth surface) contact angles, respectively. Even assuming a decrease in dentin surface energy by the exposure of collagen fibers, water contact angles become smaller because of an increase in surface roughness (13). The peritubular dentin, as above stated, is dissolved away upon etching and tubule lumens are enlarged, resulting in a much more porous dentin (13,19,21). These morphological changes could also produce a decrease in water observed contact angle by capillary action because the tubules are normally filled with fluid and naturally saturated (8).

When both surfaces were NaOClaq solution treated, dentin roughness increased in both dentins, and wettability increased only on deep dentin when contact angle was measured with water. Acid etching produces a collapse of collagen that might be due to the actions of the surface tension air forces at the air-liquid interface, which would exert a powerful force, causing the collagen matrix to flatten (22). NaOCl is a well-know nonspecific proteolytic agent demonstrating effective removal of organic components from biological materials at room temperature (7,9). After sodium hypochlorite treatment, an increase in wettability is expected, because deproteinization leads to a mineralized, naturally hydrophilic surface (16), and primarily further increases the roughness (23). NaOCl treatment removed the exposed collagen fibers, and the width of the tubular apertures became bigger for deep dentin that had been NaOCl treated (8). The removal of collagen results in dentin surface similar to those of etched enamel (17). Rougher surfaces have wider contact areas available for bonding, and also provide an increased surface free energy in comparison to flatter or smoother ones (24).

On superficial dentin, mean observed contact angles with water were not statistically different, in accordance with previous studies (13). There are morphological and chemical differences between the superficial and deep dentin structure that could account for this variance. The relative number of exposed tubules, the area of peritubular dentin, and the area occupied by intertubular dentin vary dramatically, depending on the depth of dentin being observed. In addition, Pashley showed that tubule lumen diameters are about 2 µm wider in deep dentin compared to those of superficial dentin (25). The rationale behind this conclusion is that there are more tubules per unit area from which lateral diffusion of resin can occur, in addition to vertical diffusion into the demineralized intertubular dentin from the surface. These differences in water observed contact angle between superficial and deep dentin are maintained after acid etching and NaOClaq treatment of dentin, because morphological differences do not change after these dentin surface treatments. However, deep dentin is more wettable with water compared to superficial dentin after etching and NaOClaq solution application of acid conditioned dentin (13).

In the present investigation, mean contact angle in acid etching groups increased when Prime & Bond NT resin was tested and compared with water observed contact angle ( Table 1). This difference can be explained by the increase in the intertubular dentin as inside the open tubules (21) which facilitate the water spreading on the dentin surface. Similar resin contact angle values were found in acid-treated dentin as in NaOClaq treated demineralized dentin. These two facts are probably due to the presence on nanofiller in the PB NT formulation. Filled resins are viscous and may not easily form a thin film (26). Only low amounts of filler are appropriate in filled adhesives, so as not to compromise the wetting of the bonding substrate due to both the high viscosity and their unfavorable surface area to weight ratio (27). Furthermore, the size of the filler particles is a key factor enabling the filled resin to penetrate into dentin tubules. Nevertheless, in spite of the small size of the PB NT particles (8 nm), debate still exist as to whether these particles can actually infiltrate demineralized collagen networks (27), or collagen-depleted dentin. Tam et al. found that these particles did not appear to penetrate into dentinal tubules (28); instead, the resin component diffused preferentially, by capillary action. Other researchers (18,26) observed the same phenomenon with PB NT, and suggested that filler particles congregating at the surface prevented efficient infiltration of demineralized dentin.

PB NT is an acetone/based etch&rinse adhesive that contains an acidic phosphonated monomer (PENTA: dipentaerythritol pen-taacrylate monophosphate), which also interacts with the calcium ions left on dentin surface (4) even after collagen removal (29). This molecule belong both to the group of functional and cross-linking monomers, and some of these monomers will readily hy-drolyze upon admixture with water and form separate functional monomers (27). H3PO4 and H3PO4 + NaOCl dentin treatments lead to an increase in water content as much in the intertubular dentin as inside the open tubules (8). The failure in removing all residual water entrapped in the deepest regions of demineralized and demineralized and NaOClaq treated dentin induces the formation of poorly polymerized polymer chains (6). In addition, acetone has a very good water-removing capacity, because of its high dipole moment and excellent evaporation capacities (30). However, it is not able to re-expand shrunken demineralized collagen, considering the low H-bonding capacity (27), and this may also explain the absence of difference between phosphoric etching and collagen removal surface treatment.

On the other hand, NaOCl, apart from being an effective deproteinizing agent, is also a potent biological oxidant (31), and in aqueous solution superoxide radicals -O2- are formed (3) and hydrogen-bonding interactions may also occur between side chains with residues of the organic matrix (19).

The results of the present study support the previous findings (18,32) in order that the qualitative and quantitative role of collagen fibers in optimizing adhesion must be questioned. The null hypothesis can only be confirmed in part. The application of a 5% NaOClaq solution for 2 min after etching resulted in a significant increase in roughness and water observed contact angle by the opening and widening of superficial and deep dentin tubules. However, the presence of nanofiller in PB NT or hydrogen-bonding interactions with residues of the organic matrix on collagen-depleted dentin might have influenced the absence of variation on adhesive contact angle measurements. Adhesive performance test and clinical studies should be performed prior to recommending the application of NaOCl on a routine basis with P&B NT as adhesive system.
